# New insights into the fascinating world of glucocorticoids: the dexamethasone-miR-342-Rictor axis in regulatory T cells

**DOI:** 10.1038/s41423-020-00598-0

**Published:** 2021-01-06

**Authors:** Frank Buttgereit, Timo Gaber

**Affiliations:** Department of Rheumatology and Clinical Immunology, Charité University Medicine, Berlin, Germany

**Keywords:** Immunology, Cell signalling

In a recent issue of Immunity, Kim et al. reported previously unknown effects of glucocorticoids on murine regulatory T (Treg) cells, and this report added another very interesting perspective to the already very diverse body of knowledge about these important hormones and therapeutics compared to the body of knowledge about other clinically used drugs.^1^ Treg cells are important regulators of the immune response in autoimmune and allergic inflammation. Both human and murine Treg cells are characterized by high expression of the cytokine IL-2 receptor α chain (CD25) and expression of the transcription factor Foxp3, which is necessary for the development, function and stability of these cells. The main function of Treg cells is the regulation or suppression other immune cell functions, such as the activation, proliferation, and cytokine production of CD4+ T cells, CD8+ T cells, B cells, and dendritic cells. In this way, Treg cells control the immune response to antigens, including toxins, chemicals, bacteria, viruses, or other substances, that come from outside the body as well as the immune response to self-antigens, thereby helping to prevent autoimmune disease. However, little is known about the effects of glucocorticoids on these cells. This lack of understanding is initially surprising because Treg cells were identified many years ago, and research in the field of glucocorticoids is very intensive due to the immense clinical importance of these therapeutics. Given this background, the new insights gained represent an important enrichment of our knowledge about both Treg cells and the mechanisms of glucocorticoid action and may help to identify new ways to optimize the benefit-risk ratio of these important drugs.

**Fig. 1:**
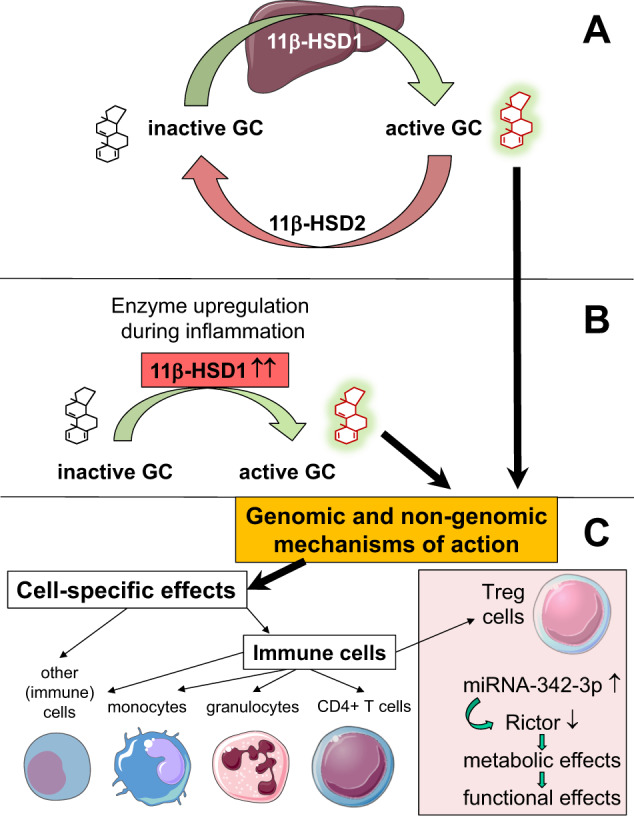
Determinants of the therapeutic effects of glucocorticoids. **A** At the systemic level, shuttling between active and inactive forms of circulating glucocorticoids (GC) is mediated by the actions of 11β-HSD (hydroxysteroid dehydrogenase) enzymes. **B** The peripheral upregulation of 11β-HSD1 in inflamed regions locally reactivates inactive glucocorticoid molecules. **C** Both systemic and local prereceptor metabolism determines the strength of genomic and nongenomic effects in a cell-specific manner. In Foxp3+ regulatory T cells, the anti-inflammatory roles of glucocorticoids are mediated via a miR-342-dependent mechanism.^1^ Medical images adapted from Servier Medical Art, http://smart.servier.com/, licensed under a Creative Commons Attribution 3.0 Unported License

For more than 70 years, glucocorticoids have represented an integral part of modern clinical medicine.^2^ In rheumatology alone, glucocorticoids are mentioned in many current recommendations, e.g., for the management of rheumatoid arthritis, large vessel vasculitides, polymyalgia rheumatica and giant cell arteritis. In sharp contrast to this very high clinical significance, the exact mechanisms of action are understood in less detail.^3^ However, we are constantly learning more. Only recently, very interesting findings about the contribution of prereceptor glucocorticoid metabolism by the hydroxysteroid dehydrogenase enzyme 11β-HSD1 to anti-inflammatory mechanisms were published. Fenton et al. reported that glucocorticoid molecules that have undergone systemic inactivation are peripherally (i.e., locally in the inflammation area) reactivated by the enzyme 11β-HSD1 to exert strong anti-inflammatory effects. The nicotinamide adenine dinucleotide phosphate (NADPH)-dependent enzyme 11β-HSD1 is upregulated in inflammatory regions and causes local glucocorticoid activation.^4^ This phenomenon facilitates binding to cytosolic glucocorticoid receptor α to trigger genomic effects (Fig. 1).

In addition to these unique properties of prereceptor metabolism, the second level of complexity of the effects of glucocorticoids arises from the variety of genomic and nongenomic effects induced by glucocorticoids (Fig. 1).^2^ Due to the lack of space, the current state of knowledge cannot be discussed here, but an updated tabular summary can be found in Buttgereit.^3^

Third, the great variety and complexity of the effects of glucocorticoid are determined by the fact that these effects are highly dependent on cell type (Fig. 1). In a recent study, Franco et al. aimed to develop a greater understanding of how glucocorticoids affect distinct cell subsets.^5^ To this end, these authors studied the genome-wide transcriptional response to glucocorticoids in nine primary human hematopoietic and nonhematopoietic cell types, namely, B cells, CD4+ T cells, monocytes, neutrophils, endothelial cells, osteoblasts, myoblasts, fibroblasts, and preadipocytes, all of which were obtained from healthy donors. As a key result, the authors revealed a strong and previously undescribed cell type-specific transcriptional response to glucocorticoids. In detail, the authors found that the glucocorticoid methylprednisolone induces cell-specific differences in the regulation of genes and signaling pathways. These differences affected more than 9000 unique genes, which corresponds to ~17% of the human transcriptome. Cell-specific observations such as this have the potential to facilitate the development of more selective immunoregulatory therapies.

In this context, the work of Kim et al. now makes a decisive contribution.^1^ The authors started by demonstrating Foxp3+ Treg cells to be necessary for the glucocorticoid-induced inhibition of inflammatory reactions. The data revealed, in mouse models of experimental autoimmune encephalomyelitis and allergic airway inflammation, that dexamethasone was no longer able to inhibit inflammation in the absence of Treg cells. In a series of elegant follow-up experiments, the authors first showed that dexamethasone administered at the beginning of the disease effectively attenuated the signs of inflammation or clinical symptoms. However, when Treg cells were depleted, dexamethasone was no longer able to exert these effects. In turn, the transfer of Treg cells was shown to restore the therapeutic effect of dexamethasone treatment. Consistent observations were made in Treg cell-specific glucocorticoid receptor-deficient mice. In these animals, the lack of endogenous glucocorticoid signaling in Treg cells had little impact on immune homeostasis under steady state conditions, and the course of experimental autoimmune encephalomyelitis remained unchanged despite treatment with dexamethasone. What is the underlying mechanism that explains these interesting and clinically very relevant observations? The authors reported that dexamethasone triggered a signal specifically in Treg cells that mediated the therapeutic effects. In this context, the authors coined a new term, namely, the identification of the ‘dexamethasone-miR-342-Rictor axis’. In detail, the authors demonstrated that dexamethasone induces miRNA-342-3p in Treg cells. MicroRNA-342 (miR-342) is a highly conserved microRNA encoded by the third intron of the Evl gene. This gene is one of the genes induced by dexamethasone in Treg cells and encodes the Ena-vasodilator stimulated phosphoprotein, an actin-associated protein involved in various processes associated with cytoskeleton remodeling and cell polarity.^1^ The general function of microRNAs, which are small noncoding RNA molecules, is the modification of both the stability and translation of mRNAs, which ultimately leads to RNA silencing and posttranscriptional regulation of gene expression. Specifically, for Treg cells, the authors reported that Rictor, an mTOR complex 2 molecule, served as a potential target of miR-342-3p. Indeed, the modulation of miR-342 and Rictor expression could influence the metabolic profile and thus the suppressive function of Treg cells. It was concluded that dexamethasone induced the miR-342-3p-mediated downregulation of Rictor in Treg cells, which preserved oxidative phosphorylation (OXPHOS) metabolic programming and limited the glycolytic pathway, thereby mediating anti-inflammatory effects.

Examining the metabolism of Treg cells in detail certainly would have been slightly beyond the scope of this paper. Therefore, the authors end the article with the conclusion that the dexamethasone-mediated downregulation of Rictor via miR-342-3p enhanced the suppressive functions of Treg cells by supporting OXPHOS and inhibiting the enhancement of glycolytic pathways. Nevertheless, there is much to be said on this subject because, in recent years, a new field called *immunometabolism* has emerged, which focuses on changes in intracellular metabolic pathways in immune cells that alter their function.^6^ We and others have recently described the complex interplay between metabolic reprogramming and immunity.^6,7^

The reprogramming of metabolism from OXPHOS to aerobic glycolysis (the ‘Warburg effect’) upon cell activation is a hallmark of effector T cells. Conversely, FOXP3+ Treg cells do not require high rates of glycolysis and high uptake of glucose or glutamine but mainly depend on the oxidation of mitochondrial lipids, pyruvate, and lactate.^7^ Moreover, the Treg transcription factor Foxp3 itself intervenes with Treg metabolism by downregulating glycolysis, inducing OXPHOS, and increasing the NAD:NADH ratio. Under metabolically challenging conditions, lactate impairs effector T cells through lactate dehydrogenase (LDH)-mediated NAD depletion, which can be replenished in Tregs by OXPHOS. These metabolic adaptations enable Tregs to maintain their immunosuppressive function in low-glucose, lactate-rich environments.^7^ In contrast, high rates of glucose metabolism can impair the suppressive capacity of Treg cells by inducing mTORC1, while lower levels of mTORC1 activity are still necessary for proper Treg function. In the absence of any mTORC1 function, Treg cells fail to suppress effector T cells. Conversely, the mTORC1 inhibitor rapamycin is capable of promoting the development of Tregs.^7^

When Tregs fail to properly differentiate and thus become dysfunctional, as observed in patients suffering from immunodysregulation polyendocrinopathy enteropathy X-linked (IPEX) syndrome, enhanced phosphorylation of mTORC2, but not mTORC1, can be observed. Conversely, treatment of Tregs obtained from these patients with an mTOR inhibitor restores their suppressive function.^8^ In Foxp3-deficient murine Treg cells, the regulatory function can be re-established by specifically deleting the mTORC2 adapter gene *Rictor* but not by deleting the mTORC1 adaptor gene *Rptor*.^8^ Moreover, deletion of Foxo1, the expression of which is reduced due to hyperactivation in Foxp3-mutated Tregs, impairs the reversal of IPEX syndrome in mTORC2 KO mice. Thus, inhibition of Foxo1 by mTORC2 reduces the suppressive capacity of Tregs by enhancing their glycolytic rate.^8^ Given this background, the interesting findings provided by Kim et al. complete our understanding of the reprogramming of immunometabolism from glycolysis back to OXPHOS to enhance immune suppression by targeting mTORC2 (including Rictor).^1^

In summary, the publication by Kim et al. describes carefully conducted experiments with clear and novel conclusions, but it also has limitations. First, it needs to be shown that the findings obtained in murine models can also be confirmed in human Treg cells. Second, the authors argue that the continuation of this research could lead to the identification of new therapeutic targets, for example, to better control glucocorticoid-resistant inflammation by targeting the miR-342-Rictor axis in Treg cells. However, no precise and comprehensible concept of how this idea should be implemented in the near future is discernible. Of note, the latter would be urgently needed to further and decisively improve the current benefit-risk ratio of treatments including glucocorticoids.^9,10^
